# Fulminant Disseminated Intravascular Coagulation as Initial Presentation of BRAF-Mutated Melanoma

**DOI:** 10.1155/2019/9246596

**Published:** 2019-04-09

**Authors:** Jeremy Chuang, An Uche, Rohan Gupta, Kim Margolin, Phyllis Kim

**Affiliations:** ^1^Department of Internal Medicine, Harbor-UCLA Medical Center, 1000 West Carson Street, Torrance, CA 90509, USA; ^2^Division of Hematology and Medical Oncology, Harbor-UCLA Medical Center, 1000 West Carson Street, Torrance, CA 90509, USA; ^3^Department of Medical Oncology and Therapeutics Research, City of Hope National Medical Center, 1500 E Duarte Rd, Duarte, CA 91010, USA

## Abstract

Acute disseminated intravascular coagulation (DIC) is a pathological process involving dysfunction of the coagulation cascade. In this case report, we discuss a 33-year-old woman with BRAF V600E-mutated metastatic melanoma who presented in fulminant DIC with concurrent hemorrhagic and thrombotic manifestations and discuss the patient's brief response to combination therapy. In our discussion, we highlight the current understanding of DIC and also identify opportunities for future research to elucidate the genetic aberrations in melanoma that may result in treatment resistance to combination therapy.

## 1. Introduction

Acute disseminated intravascular coagulation (DIC) is an infrequent presenting syndrome of solid tumors. Herein, we discuss a 33-year-old woman with BRAF V600E-mutated metastatic melanoma who presented in fulminant DIC with concurrent hemorrhagic and thrombotic manifestations and only a brief response to combination BRAF and MEK inhibition.

## 2. Case Report

A 33-year-old Caucasian woman presented with one day of hematochezia and hematemesis and an enlarging left lower quadrant inguinal mass over the prior six months. She had a 9 × 9 cm tender mass in the left inguinal region and a diffusely tender abdomen. Initial laboratory tests showed hemoglobin 9.9 g/dL, platelets 154K/cumm, INR 3.51, PT 35.5 seconds, PTT 35.4 seconds, serum fibrinogen < 30 mg/dL, elevated D dimer, and serum lactate 3.9 mmol/L. Serum creatinine was 0.96 mg/dL, alkaline phosphatase 51 U/L, AST 35 U/L, ALT 17 U/L, total bilirubin 1.1 mg/dL, and LDH 615 U/L. Computerized tomography scans showed multiple small pulmonary nodules, small bowel dilatation, a 6.8 cm left pelvic mass associated with peritoneal caking, and lympadenopahy in the neck, central mesentery, and inguinofemoral chain.

The patient underwent emergency laparotomy for a hemoperitoneum and small bowel intussusception secondary to a small bowel metastasis of melanoma (later determined to have BRAF v600E mutation). On postoperative day 3, she developed dusky discoloration of the nose and several digits and was treated with unfractionated intravenous heparin. Her course was further complicated by intra-abdominal hematoma, necrotic bowel secondary to microthrombi requiring resection, extensive limb necrosis requiring amputations, and acute tubular necrosis requiring hemodialysis.

A primary cutaneous melanoma was not identified. The patient received dabrafenib and trametinib and experienced prompt resolution of DIC and improvement of renal function. Unfortunately, control of malignancy was brief (<4 weeks), and she died of disease without recurrent DIC.

## 3. Discussion

Although several malignancies are associated with DIC, there are limited case reports of acute DIC as the initial presentation of metastatic melanoma (Table 1) [1–3]. Tissue factor, a potent procoagulant, is generated by tumor cells and inflammatory cells as well as by tissue necrosis and endothelial damage. Cancer procoagulant (CP), which directly activates factor X, has been associated with metastatic melanoma cells [4]. Tumor necrosis factor, interleukin-6, and other malignancy-associated proinflammatory cytokines may also contribute [5]. In our patient, the triggers for DIC could have been tissue damage from widespread peritoneal metastases overexpressing TF/CP or highly aggressive tumor with increased cell turnover, which would be consistent with the remarkably brief period of disease control using combined MAP kinase inhibition. It is likely that this patient's melanoma had from the outset a set of mutations or a pattern of gene expression associated with highly aggressive behavior, DIC, and resistance to mutation-targeted therapy.

## Figures and Tables

**Table 1:** Case reports of metastatic melanoma presenting with DIC.

Source	Clinical presentation	Diagnosis	Treatment course	Citation
Bhattacharyya et al.	62-year-old woman presenting with hematuria and DIC 2 months after she was diagnosed with BRAF-mutated metastatic melanoma.	BRAF-mutated metastatic melanoma	DIC improved with vemurafenib.	[1]
Lepelley-Dupont et al.	61-year-old woman with history of metastatic melanoma presenting with acute hemorrhagic shock with evidence of DIC.	Metastatic melanoma	Dacarbazine was started; however, patient expired secondary to hemorrhagic shock.	[2]
Schlaeppi et al.	37-year-old man with history of excised melanoma 6 years ago found to have diffuse metastatic disease of the liver and DIC.	Metastatic melanoma	DIC resolved with dacarbazine, vinblastine, and cisplatine.	[3]
